# Analysis of brain region-specific co-expression networks reveals clustering of established and novel genes associated with Alzheimer disease

**DOI:** 10.1186/s13195-020-00674-7

**Published:** 2020-09-02

**Authors:** Daniel Lancour, Josée Dupuis, Richard Mayeux, Jonathan L. Haines, Margaret A. Pericak-Vance, Gerard C. Schellenberg, Mark Crovella, Lindsay A. Farrer, Simon Kasif

**Affiliations:** 1 Bioinformatics Graduate Program, Boston University, Boston, MA USA; 2 Department of Medicine (Biomedical Genetics E200), Boston University School of Medicine, 72 East Concord St., Boston, MA 02118 USA; 3 Department of Biostatistics, Boston University School of Public Health, Boston, MA USA; 4 Department of Neurology and Sergievsky Center, Columbia University, New York, NY USA; 5 Department of Population and Quantitative Health Sciences, Case Western Reserve University, Cleveland, OH USA; 6 Hussman Institute for Human Genomics, University of Miami Miller School of Medicine, Miami, FL USA; 7 Department of Pathology and Laboratory Medicine, University of Pennsylvania, Philadelphia, PA USA; 8 Department of Computer Science, Boston University, Boston, MA USA; 9 Department of Neurology, Boston University School of Medicine, Boston, MA USA; 10 Department of Ophthalmology, Boston University School of Medicine, Boston, MA USA; 11 Department of Epidemiology, Boston University School of Public Health, Boston, MA USA; 12 Department of Biomedical Engineering, Boston University, Boston, MA USA

**Keywords:** Alzheimer disease, Gene network analysis, Brain regions, Genome-wide association study, *EPS8*, *HSPA2*

## Abstract

**Background:**

Identifying and understanding the functional role of genetic risk factors for Alzheimer disease (AD) has been complicated by the variability of genetic influences across brain regions and confounding with age-related neurodegeneration.

**Methods:**

A gene co-expression network was constructed using data obtained from the Allen Brain Atlas for multiple brain regions (cerebral cortex, cerebellum, and brain stem) in six individuals. Gene network analyses were seeded with 52 reproducible (i.e., established) AD (RAD) genes. Genome-wide association study summary data were integrated with the gene co-expression results and phenotypic information (i.e., memory and aging-related outcomes) from gene knockout studies in *Drosophila* to generate rankings for other genes that may have a role in AD.

**Results:**

We found that co-expression of the RAD genes is strongest in the cortical regions where neurodegeneration due to AD is most severe. There was significant evidence for two novel AD-related genes including *EPS8* (FDR *p* = 8.77 × 10^−3^) and *HSPA2* (FDR *p* = 0.245).

**Conclusions:**

Our findings indicate that AD-related risk factors are potentially associated with brain region-specific effects on gene expression that can be detected using a gene network approach.

## Background

Neurodegenerative diseases, such as Alzheimer disease (AD), Parkinson disease (PD), Huntington disease (HD), and amyotrophic lateral sclerosis, impair or damage neurons. Although many sub-cellular similarities between neurodegenerative diseases have been identified [1], the regional differences between them are quite profound [2–5]. For example, neuronal cell death from HD is primarily localized to the basal ganglia, whereas both AD and PD result in cell death throughout the brain [5]. Furthermore, PD causes the most severe cell death in the substantia nigra [2] whereas AD most heavily affects the hippocampus, the frontal cortex, and the temporal lobe [4]. These studies highlight the importance of studying gene expression signatures and relationships of AD-associated genes in different brain regions. For instance, an increased correlation in gene expression among two AD-associated genes in the brain structures such as the cortex as compared to other brain regions suggests either a functional relationship or cell/sub-region-specific expression biases towards cell types where the disease tend to originate or progress most rapidly.

Altered functional connectivity between brain regions has been demonstrated for several neuropsychiatric diseases including schizophrenia, depression, and AD using functional magnetic resonance imaging [6–8]. Brain imaging and neuropathological studies indicate that the hippocampus, which has a role in memory formation, is one of the first structures showing a marked neuronal loss in AD and, compared to other regions, suffers the largest relative reduction in volume by the latter stages of the disease [9]. Regional specificity is also evident by longitudinal patterning of the AD-related tau and amyloid-β proteins that aggregate into neurofibrillary tangles and senile plaques, respectively [10]. In the early stages of AD, a small number of tangles typically form in the brain stem and then spread aggressively to most of the cerebrum by the latest stage [11, 12]. Amyloid plaques form in the opposite pattern, beginning primarily in the outer cortex and spreading inward and then to the brain stem [10]. Notably, very few protein aggregates form in the cerebellum even at the most severe stages of AD.

Differences in AD severity between the regions of the brain may be a consequence of a variety of factors. One such factor is the tissue-specific expression patterns of genes throughout the body, which is a relevant consideration for the human brain given its vast complexity and compartmentalization [13]. An additional factor may also be the changing cell type fractions observed between major regions of the brain [14, 15]. Large-scale multi-omic approaches have been able to assist in understanding these complicated roots of neuropsychiatric disease [16, 17]. Furthermore, they have been able to identify novel disease-related gene candidates [18–20].

In this study, we integrated network-based correlation methods with existing genome-wide association study (GWAS) data and gene expression data derived from the brain in order to identify additional AD-related genes using a network methodology. In addition to identifying several novel biologically relevant genes for AD, we show that the strength of the correlations among previously established AD genes increases when the networks are restricted to the sub-regions of the brain that are most impacted by AD.

## Methods

### Acquisition of GWAS data and curation of AD genes

We obtained summarized results from a GWAS for AD risk conducted using 16,175 AD cases and 17,175 controls of European ancestry that were obtained as previously described [21, 22]. Association evidence with each gene was derived from *p* values for the association with individual single nucleotide polymorphisms (SNPs) corrected for multiple testing using an approximation that has been shown to be a conservative adjustment for recombination hotspots, linkage disequilibrium, and gene size [23]. This correction can be expressed as:

$$ {P}_g^{{\mathrm{Gene}}^{\prime }}=1-{\left(1-{P}_g^{\mathrm{BestSNP}}\right)}^{\frac{N+1}{2}} $$

where *N* is the number of SNPs existing within a gene. Analyses for this study were also predicated on a group of reproducible AD (RAD) genes which were previously curated from the literature [21].

### Acquisition, labeling, and processing of brain expression data

Measurements of gene expression in the human brain were acquired from the Allen Brain Atlas (ABA). This database contains microarray data from 3702 single tissue samples extracted from six neuropathologically healthy brains (ages 24, 26, 31, 39, 49, and 57). Data derived from each sample consist of an expression vector containing expression measurements from 45,000 probes in the extracted tissue. Each sample was annotated at three different levels of granularity, which are defined for the purpose of this study as low-, mid-, or high-level structures in terms of region specificity. Principal components (PCs) of the expression vectors across all samples were computed using the prcomp method from the R programming package [24]. Then, each sample was annotated according to the brain region based on the hierarchical labeling scheme described above. A scatterplot of the first and second PCs was produced to ascertain whether the expression vectors of samples displayed batch effects related to either the region or the brains from which samples were derived.

### Determining gene expression within each brain region

Because some genes are queried by multiple probes, the mean expression of all probes mapping to each gene was computed, resulting in a gene × sample expression matrix. The expression of each gene in each brain region was adjusted using a mixed effect model approach to account for repeated sampling of both individual brains and regions in the ABA dataset [25]. Mixed effect model specification is contained in Additional file 1.

### Construction of region-specific brain co-expression networks

We constructed a co-expression network based upon correlations between all pairs of genes across the cerebrum, cerebellum, and the brain stem. In this instance, each node of the network is a gene, and each edge between genes is the absolute value of the Pearson correlation coefficient between a pair of genes. Due to the high impact of AD on the cerebrum, two additional correlation networks were created by subdividing the cerebrum into two non-overlapping subsets of regions based upon the relative time point in the course of the disease the region typically displays AD-related protein aggregation [26]. In total, there were 79 regions of the cerebrum in the ABA dataset that showed some neuropathological evidence of AD at Braak stage 1, which we refer to as the early-stage regions, and 37 regions of the cerebrum that showed more pronounced AD pathology at Braak stage 3, which we refer to as late-stage regions. Correlations between all gene pairs were computed separately for early- and late-stage regions. In order to compare correlations between sets of genes of interest across networks, we normalized the correlations within each network. For this, we applied a novel metric, referred to as median ranking by correlation (MRC), that is derived using a “leave-one-out” strategy to normalize the distribution of correlations into a uniform distribution of ranks that is comparable across networks. Details of the MRC procedure are provided in Additional file 1.

### Verifying consistency of gene rankings across correlation networks

Due to the small number of brain specimens in the ABA dataset, we tested the consistency of gene rankings by constructing a gene × gene correlation network for each brain. This procedure uses the same correlation approach described above, except that the expression levels of genes in each region were determined based on the measurements from a single brain at a time. Next, each non-RAD gene was ranked by its correlation to the RAD seed genes within each of the six individual correlation networks. Finally, a Kendall Tau rank correlation matrix was derived based upon all possible combinations of these six ranked lists.

### Network-based ranking of novel AD genes

Based on the observation that the RAD genes tend to be highly correlated, we hypothesized that other genes showing a high correlation with established AD genes are likely to be AD-related genes. Therefore, a summed absolute Pearson correlation with all the RAD genes was computed for each non-RAD gene. Next, a percentile rank of each non-RAD gene-based upon these sums was computed and converted to *Z*-scores, which we refer to as network scores. If *N* genes are ranked, then the percentile rank for each gene is percentile = (rank)/(*N* + 1), ranging from 0 to 1. These percentiles form a uniform distribution, which are converted to *Z*-scores using qnorm(Percentile, lower.tail = F) in R. These network scores were then combined with genetic association *Z*-scores derived by a GWAS for AD risk including approximately 30,000 individuals using the Stouffer method implemented [27] in the meta-analysis tool METAL that was modified to equally weight both scores [28]:

$$ {Z}_{\mathrm{combined}}=\frac{0.5\times {Z}_{\mathrm{gwas}}+0.5\times {Z}_{\mathrm{network}}}{\sqrt{0.5^2+{0.5}^2}} $$

Further ranking was performed by integrating phenotypic information from gene orthologs in Flybase to focus on genes which when knocked out in a model organism result in an AD-related phenotype including premature aging, defective memory, defective aging, and oxidative stress [29]. We also included genes which have external experimental evidence for influencing AD-related processes in human cell lines and brain. Linking fly and human gene orthologs was accomplished using the Drosophilia RNAi Screening Integrative Ortholog Prediction Tool (DIOPT) [30]. This robust consensus mapping approach has been utilized as a functional validation strategy in studies of neurologically relevant phenotypes [31]. The significance of network scores was determined based on the false discovery rate (FDR).

## Results

**Fig. 1:**
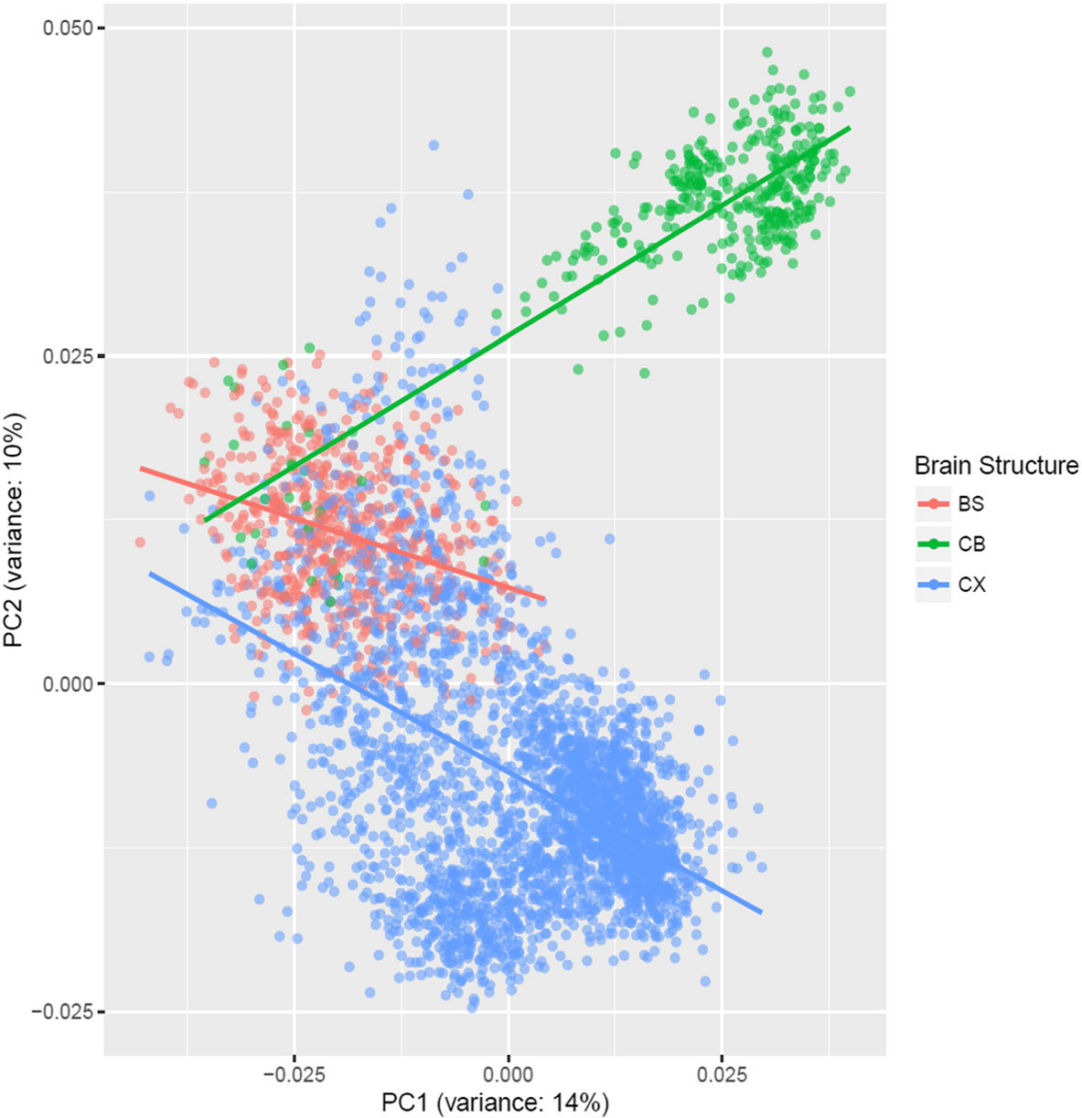
Principal component analysis of expression vectors indicates sample clustering for high-level brain structures. Principal components were computed for all samples in the dataset. There was no evidence of clustering for mid-level brain structure. CX, cerebrum; BS, brain stem; CB, cerebellum

**Fig. 2:**
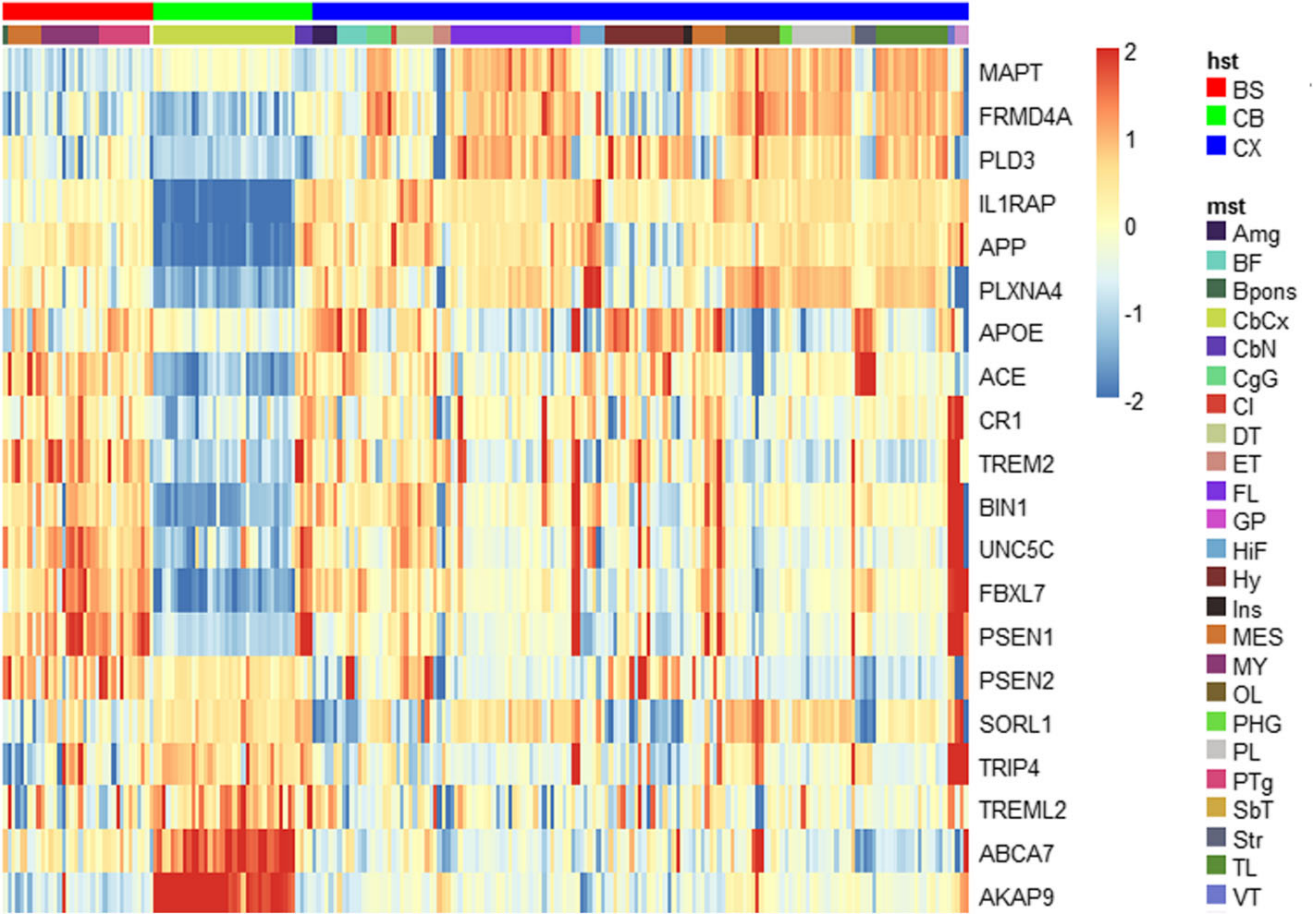
Expression of RAD genes is region-specific. Heatmap shows the expression patterns in the brain high-level structures (hst) and mid-level structures (mst) for 20 RAD genes including 10 of the most well-established AD genes (i.e., *APOE*, *APP*, *PSEN1*, *PSEN2*, *CR1*, *BIN1*, *SORL1*, *ABCA7*, *MAPT*, *TREM2*) and 10 others chosen randomly from the total set of 52 RAD genes in Table 1. Patterns for the other 32 genes were similar but not shown to improve visualization. The strength and pattern of the expression are color-coded according to the scheme shown on the right of the heatmap with red indicating increased expression and blue indicating decreased expression. BS, brain stem; CB, cerebellum; CX, cerebrum; Amg, amygdala; BF, basal forebrain; Bpons, basis pontis; CbCx, cerebellar cortex; CbN, cerebellar nucleus; CgG, central gray, gamma; CI, claustrum; DT, dentate nucleus; ET, epithalamus; FL, frontal lobe; GP, globus pallidus; HiF, hippocampal fissure; Hy, hypothalamus; Ins, insula; MES, mesencephalon; MY, myelencephalon; OL, occipital lobe; PHG, parahippocampal gyrus; PL, paralemniscal nucleus; PTg, pedunculotegmental nucleus; SbT, subthalamus; Str, subiculum, transition area; TL, temporal lobe; VT, ventral tegmental area

The principal component analysis revealed a potential batch effect in the six brain samples with respect to the gene expression in the three high-level brain structures (Fig. 1), noting that this analysis does not account for the non-independent gene co-expression. Further analysis revealed that most RAD genes tended to have fairly static expression in the cerebellum and brain stem regardless of the changes in the mid-level structure (Fig. 2). However, the expression for several of these genes appears more variable across the mid-level structures in the cerebrum.

Comparison of the co-expression of RAD genes across the high-level brain regions revealed higher correlation ranks (CRs) in the cerebrum (0.748) than in the brain stem (0.648) and cerebellum (0.574, Table 1). These differences appear to be due largely to a few genes including *APOE* and *MAPT* which showed much greater co-expression in the cerebrum (CR = 0.745 and 0.863, respectively) than in the cerebellum (CR = 0.280 and 0.542, respectively) and brain stem (CR = 0.216 and 0.337, respectively). Surprisingly, the CR for *APP* was much higher in the cerebellum (0.99) than in the brain stem (0.505) and cerebrum (0.376). Multiple RAD genes including *PSEN2*, *EPHA1*, *LMX1B*, *TPBG*, *CLU*, *AKAP9*, *ZNF804B*, *PDGFRL*, and *ABCA7* were not meaningfully co-expressed with other RAD genes in any of the structures.

**Table 1:** Ranked correlations (RC) of RAD genes in the cerebrum, cerebellum, and brain stem. Genes are ordered according to the variance (highest to lowest) of their CR across the three structures

Gene	Percentile ranking by correlation		
	Brain stem	Cerebellum	Cerebrum
*ZCWPW1*	0.991	0.080	0.783
*TRIP4*	0.997	0.226	0.772
*SORCS1*	0.076	0.602	0.807
*OSTN*	0.753	0.196	0.858
*PLXNA4*	0.182	0.601	0.854
*SORCS2*	0.785	0.136	0.605
*CASP8*	0.222	0.720	0.853
*AKAP9*	0.541	0.877	0.214
*APP*	0.505	0.990	0.376
*ABCG1*	0.392	0.325	0.897
*TP53INP1*	0.880	0.394	0.968
*PFDN1*	0.557	0.998	0.406
*COBL*	0.902	0.323	0.662
*APOE*	0.216	0.280	0.745
*SORCS3*	0.134	0.376	0.706
*EPHA1*	0.219	0.739	0.294
*ACE*	0.778	0.667	0.249
*CR1*	0.068	0.344	0.603
*MAPT*	0.337	0.542	0.863
*KCNMB2*	0.021	0.530	0.401
*CLU*	0.732	0.582	0.219
*PDGFRL*	0.634	0.395	0.126
*PLD4*	0.948	0.463	0.770
*SORL1*	0.935	0.462	0.612
*CD2AP*	0.351	0.691	0.772
*GALNT7*	0.390	0.823	0.699
*SLC10A2*	0.329	0.224	0.640
*PILRA*	0.601	0.896	0.494
*CASS4*	0.040	0.204	0.438
*PLD3*	0.536	0.355	0.752
*MS4A6A*	0.603	0.623	0.934
*C1QTNF4*	0.804	0.714	0.451
*MS4A4A*	0.728	0.499	0.859
*ABI3*	0.685	0.567	0.908
*NCR2*	0.776	0.664	0.975
*UNC5C*	0.797	0.517	0.765
*PTK2B*	0.688	0.979	0.899
*MEF2C*	0.812	0.695	0.986
*ABCA7*	0.082	0.338	0.095
*LMX1B*	0.003	0.078	0.276
*TREM2*	0.793	0.820	0.963
*ECHDC3*	0.805	0.703	0.670
*BIN1*	0.663	0.798	0.724
*PSEN2*	0.242	0.256	0.356
*CD33*	0.876	0.832	0.950
*ZNF804B*	0.073	0.141	0.162
*PICALM*	0.912	0.922	0.989
*HLA-DRB5*	0.973	0.910	0.986
*PSEN1*	0.879	0.924	0.866
*INPP5D*	0.937	0.987	0.982
*TPBG*	0.220	0.240	0.249
*PLCG2*	0.943	0.963	0.945

**Fig. 3:**
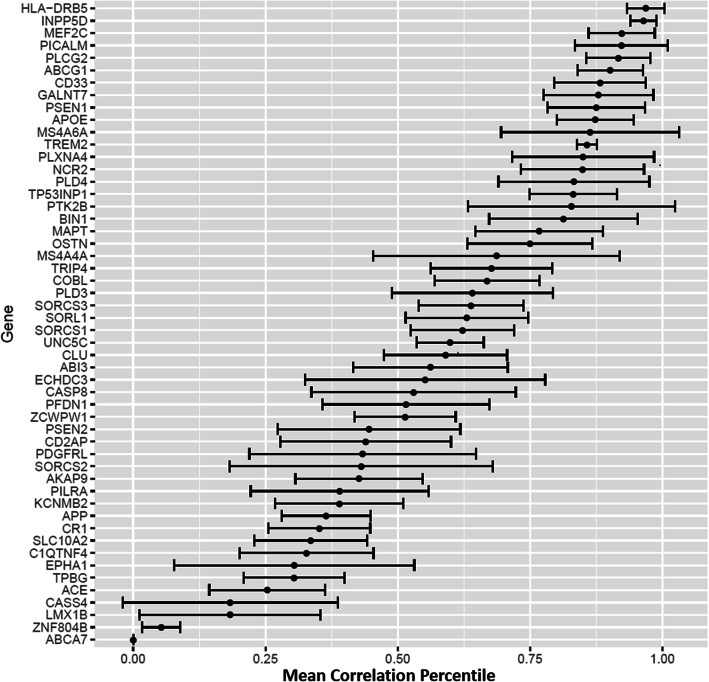
Mean rank correlations for RAD genes. A correlation network of the cerebrum was constructed for each of the six brains. The mean correlation and 95% confidence interval are shown for each RAD gene averaged across the six brains

**Fig. 4:**
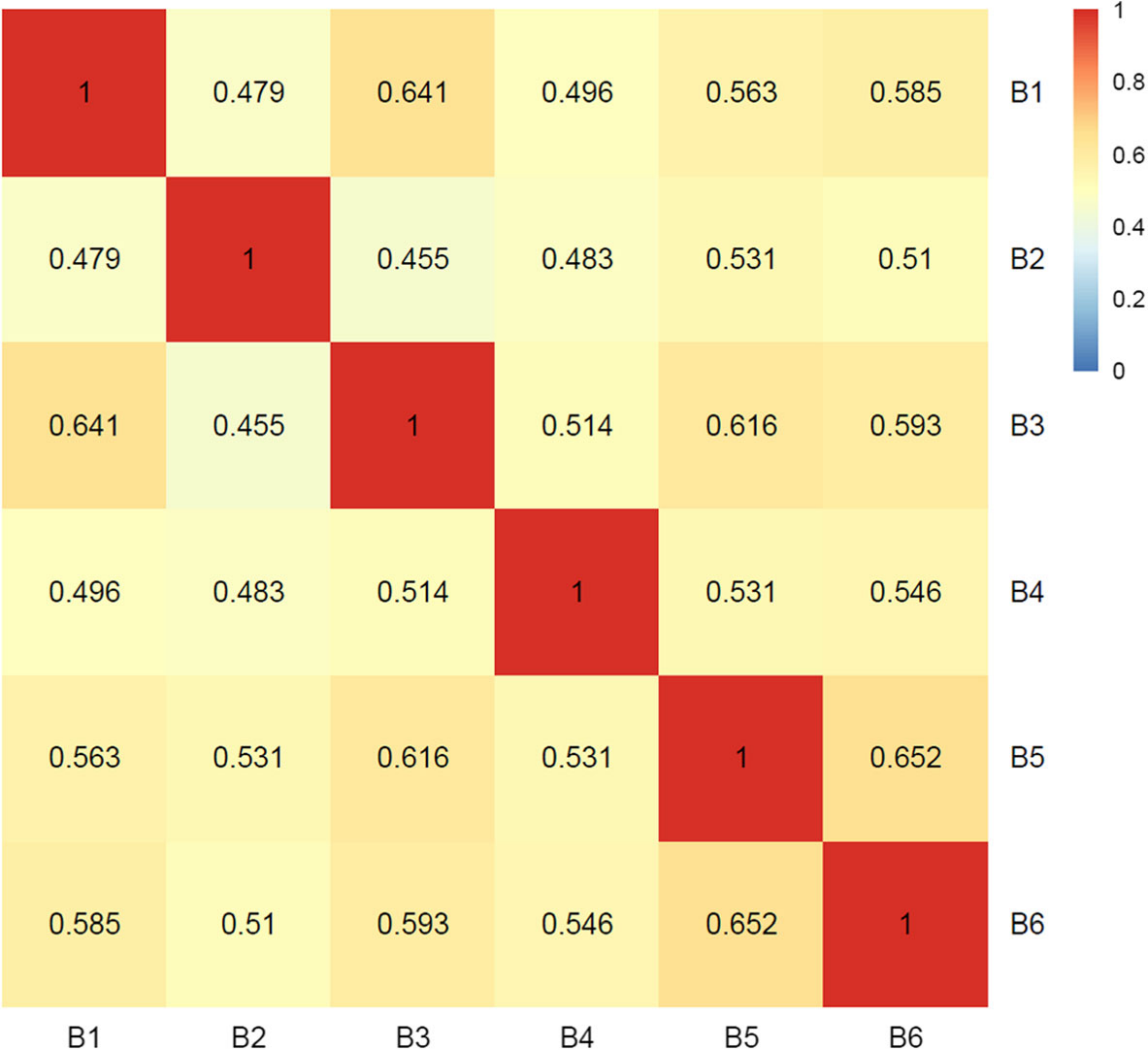
Pairwise correlations of the RAD co-expression networks among brain samples. Rankings of all non-RAD genes were derived for each of the six individual networks using the RAD genes as the seed genes. The Kendall Tau rank correlation was then computed between gene rankings using each possible pairing of networks

The MRC of the RAD genes was appreciably and nearly significantly higher for the late-stage network (0.733) than the early-stage region network (0.615, Wilcoxon signed rank *p* = 0.052), but the CRs for many individual genes including *APOE*, *APP*, and *MAPT* were similar across these two networks (Table 2). The comparison of correlation networks in the cerebrum constructed for each individual showed that RAD genes tend to have low variability in CR among individuals within this dataset (Fig. 3). The patterns of co-expression across the individual brains are moderately high and consistent with the CR values between 0.455 and 0.652 (Fig. 4).

**Table 2:** Ranked correlations (RC) of RAD genes in early- and late-stage correlation networks

Gene	Percentile ranking by correlation	
	Early	Late
*UNC5C*	0.127	0.997
*TP53INP1*	0.093	0.931
*ZCWPW1*	0.156	0.979
*ABCG1*	0.192	0.956
*PLD4*	0.233	0.963
*KCNMB2*	0.954	0.269
*ABCA7*	0.831	0.180
*CLU*	0.822	0.235
*TPBG*	0.735	0.163
*CASS4*	0.202	0.774
*SLC10A2*	0.284	0.851
*ECHDC3*	0.951	0.396
*INPP5D*	0.363	0.874
*AKAP9*	0.647	0.195
*SORL1*	0.975	0.529
*SORCS1*	0.865	0.419
*LMX1B*	0.588	0.158
*CASP8*	0.425	0.851
*OSTN*	0.080	0.505
*NCR2*	0.492	0.888
*SORCS3*	0.482	0.864
*PICALM*	0.640	1.000
*C1QTNF4*	0.105	0.428
*TRIP4*	0.619	0.896
*PSEN1*	0.712	0.984
*PSEN2*	0.594	0.380
*PILRA*	0.489	0.289
*ABI3*	0.671	0.867
*PLCG2*	0.984	0.794
*ACE*	0.065	0.249
*APOE*	0.987	0.807
*PTK2B*	0.756	0.579
*PDGFRL*	0.111	0.272
*SORCS2*	0.481	0.641
*MS4A6A*	0.728	0.884
*MS4A4A*	0.881	0.727
*TREM2*	0.849	0.999
*GALNT7*	0.737	0.882
*EPHA1*	0.330	0.467
*CD2AP*	0.531	0.667
*HLA-DRB5*	0.847	0.948
*COBL*	0.830	0.740
*PLXNA4*	0.847	0.760
*CR1*	0.610	0.691
*PLD3*	0.584	0.647
*MEF2C*	0.800	0.739
*PFDN1*	0.596	0.657
*ZNF804B*	0.219	0.168
*APP*	0.676	0.705
*BIN1*	0.949	0.925
*CD33*	0.977	0.988
*MAPT*	0.534	0.526

In order to predict novel AD genes based upon the above observations, a network score was produced for each non-RAD gene using the cerebrum correlation network in which clustering of the RAD genes was strongest. These network scores were then combined with GWAS *Z*-scores resulting in re-ordered AD gene rankings. A normal approximation was used to evaluate the significance of the combined scores. These results were filtered using gene knockout information from Flybase to limit the focus to genes which have functional evidence for producing AD-related phenotypes. Of the remaining 654 genes after the final filtering step, there was significant evidence for two novel AD-related genes including *EPS8* (FDR *p* = 8.77 × 10^−3^) and *HSPA2* (FDR *p* = 0.245) (Table 3). Several previously reported AD genes also had high rankings but were not significant after FDR correction including *ADAM10* (FDR *p* = 0.40) and *HDAC1* (FDR *p* = 0.79). Only one of the top-ranked genes, *RCAN1*, when knocked out resulted in as many as three AD-related phenotypes in flies; however, the statistical support was modest (FDR *p* = 0.79)*.*

**Table 3:** Top-ranked genes based on the combined GWAS and network score. Genes are listed only if the phenotype induced by knockdown in flies is AD-related based on evidence of defective memory (DM), defective aging (DA), oxidative stress (OS), or premature aging

Gene name	Phenotype	One-tailed ***Z***-score			***p*** value	
		GWAS	Network	Combined	Unadjusted	FDR
*EPS8*	DM	3.16	2.78	4.20	1.34E−05	8.77E−03
*HSPA2*	DA	2.98	1.51	3.17	7.51E−04	2.45E−01
*ADAM10*	DA	2.61	1.50	2.90	1.84E−03	4.01E−01
*HSPA6*	DA	2.13	1.63	2.66	3.88E−03	6.34E−01
*CAMK2A*	DM	0.94	2.60	2.50	6.13E−03	7.91E−01
*HDAC1*	DA, OS	1.40	1.97	2.38	8.55E−03	7.91E−01
*MAPK10*	DA, OS	1.58	1.68	2.30	1.06E−02	7.91E−01
*CAT*	DA, OS	2.31	0.89	2.27	1.17E−02	7.91E−01
*FXR1*	DM	0.58	2.60	2.24	1.24E−02	7.91E−01
*CD164*	DM	0.90	2.23	2.21	1.34E−02	7.91E−01
*HSPB1*	OS	1.58	1.52	2.19	1.43E−02	7.91E−01
*FBXW7*	OS	0.66	2.34	2.12	1.69E−02	7.91E−01
*DAGLB*	OS	1.52	1.48	2.12	1.71E−02	7.91E−01
*NFE2L3*	OS	1.71	1.20	2.06	1.98E−02	7.91E−01
*MAFB*	OS	1.56	1.31	2.03	2.11E−02	7.91E−01
*ITGAX*	DM	1.67	1.20	2.03	2.11E−02	7.91E−01
*SETBP1*	DA	1.26	1.60	2.02	2.14E−02	7.91E−01
*ACHE*	DM	2.12	0.71	2.00	2.28E−02	7.91E−01
*ITGAM*	DM	1.56	1.19	1.94	2.59E−02	7.91E−01
*ITPR1*	DA	1.01	1.72	1.93	2.68E−02	7.91E−01
*HBB*	OS	2.68	0.02	1.91	2.81E−02	7.91E−01
*PLK3*	DA	2.16	0.50	1.88	2.98E−02	7.91E−01
*TRIB3*	DM	1.35	1.29	1.87	3.08E−02	7.91E−01
*RCAN1*	DA, DM, OS	1.24	1.40	1.87	3.10E−02	7.91E−01
*GABARAP*	DM	0.74	1.87	1.85	3.23E−02	7.91E−01
*NIPBL*	DM	0.72	1.90	1.85	3.24E−02	7.91E−01
*GPD1*	OS	1.54	1.06	1.84	3.26E−02	7.91E−01
*GRIN2A*	DM	0.98	1.59	1.82	3.47E−02	8.10E−01
*ITPKA*	OS	0.57	1.95	1.78	3.78E−02	8.29E−01
*DNM1*	DM	1.02	1.46	1.76	3.94E−02	8.29E−01
*PGC*	DA	1.28	1.19	1.75	4.00E−02	8.29E−01
*CIDEC*	DM	1.60	0.87	1.74	4.06E−02	8.29E−01
*TXNRD2*	DA	1.85	0.59	1.73	4.21E−02	8.34E−01
*BZW2*	DM	1.60	0.80	1.69	4.51E−02	8.67E−01
*CBX3*	DA	1.54	0.81	1.66	4.81E−02	8.91E−01
*PCNA*	OS	2.31	0.03	1.65	4.91E−02	8.91E−01

## Discussion

Previous studies using correlation or other network strategies have increased discovery and understanding of the functional roles of novel disease-related genes across many biological contexts [18, 32–34]. In this study, we applied an integrative network strategy to capture complex relationships between RAD genes across the relevant regions of the brain and to aid the discovery of novel AD-related genes. This approach entailed integration of AD GWAS data, gene expression measures in multiple brain regions, and phenotypic information (i.e., memory and aging-related outcomes) from gene knockout studies in *Drosophila* [29]. By separating the regions of the brain according to the established patterns of AD-related pathology including neurodegeneration and protein aggregation, we showed that the correlation of expression between previously established AD genes is highest in regions severely impacted by AD, noting gene expression data were derived from brains without AD pathology. In addition, we identified potential novel AD genes by numerically combining results from co-expression analysis of established AD genes and other genes in relevant brain regions with summary statistics from a large AD GWAS.

The most robust novel gene identified by our approach is *EPS8*. This gene encodes epidermal growth factor receptor substrate 8 which is involved in actin cytoskeleton regulation and is abundantly expressed in many brain regions [35]. The accumulation of filamentous actin (F-actin) is associated with tau-induced neurodegeneration in *Drosophila* and mouse tauopathy models [36]. The deletion of Eps8 in mice leads to a reduction in hippocampal synaptic plasticity and impaired cognitive performance [37]. Three genes encoding heat shock proteins (*HSPA2*, *HSPA6*, and *HSPB1*) also emerged among our top findings. Notably, *HSPA2* was also identified as related to AD in a recent network analysis in an independent dataset [38]. Heat shock proteins have a major role in handling misfolded proteins including amyloid-β [39]. Although the expression of heat shock protein genes has been well studied in AD [40], there is little evidence for the association of AD risk with polymorphisms in any members of this gene family [41].

Several other top-ranked genes in our study have directly or indirectly been linked to AD. *ADAM10* encodes disintegrin and metalloproteinase 10 which is a synaptic enzyme that has been previously shown to limit amyloid-β_1-42_ peptide formation in AD. A variant in *ADAM10* recently achieved genome-wide significance in one of the largest genetic studies of AD containing more than 95,000 individuals [42, 43]. The catalase protein encoded by *CAT* binds with amyloid and inhibition of this interaction has been reported to protect cells from toxic protein aggregation [44, 45]. Several genes in the *HDAC* family have been reported to impair memory in animal models, and inhibitors of several members of the *HDAC* gene family, including *HDCA1* identified for the first time in our study as an AD candidate gene, have been gaining support as a therapeutic approach for treating AD [46–48]. In humans, loss of *HDAC5* impairs memory function [47] and variants in *HDAC9* have been associated with a dual outcome of neurofibrillary tangles and amyloid angiopathy [49]. We also obtained mild evidence supporting a role for the gene encoding acetylcholinesterase (*ACHE*). This is a noteworthy finding in light of inconsistent and generally negative reports of association for AD with *ACHE* and related genes encoding choline acetyltransferase (*CHAT*) and butyrylcholinesterase (*BCHE*), despite the fact that AD is characterized by an extensive loss of cholinergic neurons from the basal forebrain area and the wide use of cholinesterase inhibitors to treat the early stages of cognitive decline [50]. Expression of *RCAN1*, which encodes the regulator of calcineurin 1 and the only gene when knocked out resulted in three AD-related phenotypes in *Drosophila*, is increased in AD brain [51], and overexpression of the human RCAN1.1S isoform inserted in mice promotes early age-dependent memory and synaptic plasticity deficits and mitochondrial dysregulation leading to tau pathology [52].

A major motivation for our approach was to determine if the brain region-specific effects exhibited by AD can be detected using a correlation network approach. Recent work indicates that cell type compositions of the brain regions are highly variable in aging brains, so the cross-regional analysis is able to capture important properties such as changing cell fractions that may explain why the biological symptoms of AD are not uniformly present throughout the brain [15]. The high MRC of the RAD genes in the cerebrum supports this notion, given that the cerebrum tends to be the most major structure in the brain affected by AD [4]. Further evidence for this is also provided by the low MRC of the RAD genes in the other brain regions (brain stem, cerebellum) where the effect of AD is far less severe. Notably, these patterns appear to be consistent in our study of cognitively healthy individuals (Fig. 4).

Our findings also highlight several interesting patterns among several well-established RAD genes. We observed that expression of *APOE* and *MAPT* is highly correlated with other RAD genes in the cerebrum to the other RAD genes, but much less in the cerebellum and brain stem which is consistent with our observation of the RAD gene set as a whole. While most RAD genes are not highly correlated in the cerebellum, we observed a strong correlation among a few RAD genes, most notably, *APP*, which had a high CR in the cerebellum (0.99), but not in the cerebrum (0.38). *APP* is expressed across most regions of the brain, as evidenced in the Gene Tissue Expression (GTEx) portal [53]. One possible explanation for a higher correlation of expression for a few RAD genes such as *APP* in the cerebellum is that they have an important role throughout the brain, whereas other RAD genes have a more localized role in cerebral function and health. A clearer understanding of this pattern will require a focused analysis of gene co-expression within specific regions in the cerebrum.

Interpretation of our results has several caveats. First, we analyzed a dataset that has few individuals but a high number of brain regions in which expression was measured. However, the expression patterns were consistent across individual brains in the dataset. If we had chosen instead a publicly available dataset containing a larger number of individuals but expression measurements in fewer regions, we would not have observed the high variation in the expression of the RAD genes across regions of the cerebrum. This underscores the need for larger samples of brains with expression data in more precisely defined regions. Second, the present study did not include any brains from AD individuals. Although we utilized known Braak staging to characterize regions, it is necessary to compare gene expression in the brains showing progressively severe AD pathology to determine whether the patterns observed in this study are related to AD. In addition, expression patterns for only a few genes remained significant after correction for multiple testing likely due to the relatively small sample size. Finally, because no brains from persons with AD were included in this study, we were unable to evaluate whether any of the gene co-expression patterns we identified differ between those with and without AD or are correlated with the degree of AD pathology in specific brain regions. However, the purpose of this study was to investigate whether established AD risk genes are co-expressed in the brain and whether their co-expression varies by brain region, specifically regions that are affected early in the disease compared to regions that are spared until later in the disease process. Because our findings were observed in a study of brains from relatively young individuals most of whom were autopsied several decades before typical onset of AD symptoms after age 65, our findings do provide insight about the coordinated expression of genes that are known to have a role in AD and specifically in regions of the brain that are temporally affected by the disease. Finally, the expression patterns for only a few genes remained significant after correction for multiple testing likely due to the relatively small sample size.

## Conclusions

This work establishes a strong case for many potential follow-up investigations. Analysis of the expression in more fine-grained brain regions in larger samples including individuals with pathologically confirmed AD at various stages will allow more concise conclusions about the joint influences of multiple genes on the progression of AD from preclinical to late stages. Although highly granular regional expression data from AD brains is not readily available, efforts are in progress by the AMP-AD consortium to profile the expression of various regions of AD brains [54]. Validated differences in cross-regional correlation patterns between healthy and AD brains would improve the understanding of the mechanisms underlying the progression of AD and inform strategies for developing more effective therapeutic targets.

## Supplementary information

**Additional file 1.** Mixed effect model specification and median ranking by correlation methods.

## Abbreviations

ABA: Allen Brain Atlas
AD: Alzheimer disease
BS: Brain stem
CB.: Cerebellum
CR: Correlation rank
CX: Cerebrum
FDR: False discovery rate
GWAS: Genome-wide association study
HD: Huntington disease
MRC: Median ranking by correlation
PD: Parkinson disease
RAD: Reproducible Alzheimer disease
SNP: Single nucleotide polymorphism
