# Supplementary material for: Analysis of brain region-specific co-expression networks reveals clustering of established and novel genes associated with Alzheimer disease
Source: Alzheimers Res Ther. 2020 Sep 2;12:103. doi: 10.1186/s13195-020-00674-7 (PMC7469336; doi:10.1186/s13195-020-00674-7)
Supplement: Supplementary file 1 — Additional file 1. Mixed effect model specification and median ranking by correlation methods. [file 13195_2020_674_MOESM1_ESM.docx]

Adjusting for Repeated Sampling Using Mixed Effect Models

Each gene has an expression vector, *E,* of its expression levels in each sample. Each sample has an indicator of the *region* and the *brain* it was taken from. The model specification in R is then:
*E* ~ -1 + *region +* (1 | *brain*)

The *region* coefficients of this model represent a single gene’s expression in each of the 232 low level regions. This model is used for each individual gene, to ultimately determine each gene’s expression within each of the low level regions ascertained in the ABA dataset.

Median Ranking by Correlation

Let RAD be the set of RAD genes and let G-RAD be the set of other genes.

1. For each gene G_i_ in RAD let RAD-G_i_ be the set of genes in RAD different from G_i_. For each G_i_ we compute the total sum of the correlations of Gi to the other genes in RAD(RAD-G_i_). We refer to this as SUM-COR(G_i_, RAD-G_i_). Now for each gene G_j_ in G-RAD (genes outside the RAD set) we compute the full distribution of sum of correlations to genes SUM-COR(G_j_,RAD-G_i_).
2. Let the Rank(G,RAD-G_i_) be the rank of SUM-COR(G_i_, RAD-G_i_) in the full distribution of SUM-COR(G_j_,RAD-Gi).
3. Let MRC be the median such rank.

If a gene set is clustered, the median of these rankings (MRC) of the RAD genes will exceed the expectation of 0.5.
